# Metabolic acclimation to captivity in highveld mole-rats (*Cryptomys hottentotus pretoriae*) is driven by sex-specific body mass increases

**DOI:** 10.1242/jeb.252449

**Published:** 2026-05-19

**Authors:** Jack E. Thirkell, Nigel C. Bennett, Daniel W. Hart, Chris G. Faulkes, Monica A. Daley, Craig R. White, Steven J. Portugal

**Affiliations:** ^1^ Department of Biological Sciences, School of Life and Environmental Sciences, Royal Holloway University of London, Egham, Surrey, TW20 0EX, UK; ^2^ Mammal Research Institute, Department of Zoology and Entomology, University of Pretoria, Hatfield 0028, Pretoria, South Africa; ^3^ School of Biological and Behavioural Sciences, Queen Mary University of London, E1 4NS, UK; ^4^ Department of Ecology and Evolutionary Biology, University of California, Irvine, Irvine, CA 92697, USA; ^5^ School of Biological Sciences, Monash University, Clayton, VIC 3800, Australia; ^6^ Department of Biology, The University of Oxford, Oxford, OX1 3SZ, UK

**Keywords:** Bathyergidae, Metabolic rate, Respirometry, Respiratory quotient

## Abstract

Captivity represents a profound environmental shift that can induce physiological acclimation, yet its effects on metabolic rate remain poorly resolved, particularly in subterranean mammals. African mole-rats (Bathyergidae) are frequently studied under captive conditions, despite wide variation in acclimation periods prior to metabolic assessment. Here, we tested whether prolonged captivity alters resting metabolic rate (RMR) and related physiological traits in the highveld mole-rat *Cryptomys hottentotus pretoriae*, and whether such changes are associated with body mass and sex. Using open-flow respirometry, we measured RMR, mass-specific RMR (msRMR) and respiratory quotient (RQ) in the same population of wild-caught animals assessed 7 days post-capture (wild) and again after 12 months in captivity (captive). Whole-animal RMR did not differ between wild and captive groups, nor between sexes. However, msRMR was 26.1% lower following captivity, coincident with a 28.1% increase in mean body mass. This mass gain was strongly sex specific: males increased body mass by 52.4%, whereas females showed no significant change. Despite this, sex did not independently explain variation in RMR or msRMR. Captive animals also exhibited lower RQ values than recently captured individuals, suggesting shifts in substrate utilisation or energetic state. Together, these results indicate that apparent reductions in msRMR following captivity are driven primarily by increases in metabolically inactive tissue rather than suppression of whole-animal metabolic rate. Our findings highlight the importance of accounting for captivity-induced changes in body mass and composition when interpreting metabolic data, and caution against direct comparisons between unacclimated and long-term captive animals.

## Teaser

**Summary:** Duration in captivity must be considered when interpreting physiological data and making comparisons between captive and wild animals.

## INTRODUCTION

Almost all species have behavioural and physiological mechanisms to minimise the impact of aversive stimuli. Acclimation, defined by Fregley (1996) as the coordinated phenotypic response developed by the animal to a specific stressor in the environment, is one such mechanism. Many species have been shown to physiologically acclimate when faced with a range of novel environmental conditions. For example, leaf-eared mice (*Phyllotis darwini*) from the same litter but reared at different ambient temperatures during post-weaning development exhibit different basal metabolic rates (BMR) (Cavieres et al., 2017). Animals maintained at colder temperatures had higher BMR as adults, suggesting that environmental conditions experienced during ontogeny can drive thermal acclimation of metabolic rate. Deer mice (*Peromyscus maniculatus*) have been documented to have significantly increased field metabolic rates at higher altitudes (Hayes, 1989). Furthermore, Arabian oryx (*Oryx leucoryx*) are known to reduce their resting metabolic rate (RMR) by 16% when food is restricted (Ostrowski et al., 2006). Similarly, irregularly fed king penguin chicks (*Aptenodytes patagonicus*) are more efficient at staying warm compared with regularly fed animals, equating to a near 25% reduction in RMR and thus suggesting a metabolic acclimation to food shortages (Monternier et al., 2014). Even habituation to an experimental design has been noted to cause a 30% reduction in BMR in budgerigars (*Melopsittacus undulatus*) (Jacobs and McKechnie, 2014). These examples showcase some of the many different types of conditions – thermal stress, high-altitude, food-shortage, experimental design – that have been determined to bring about change in a species’ metabolic rate, and in doing so demonstrate physiological plasticity to environmental constraints (Merchant et al., 2024a). Captivity can alter multiple elements of a species’ behaviour and physiology (O'Regan and Kitchener, 2005; Fischer and Romero, 2019). This is not limited to changes in body composition – with increases in metabolically inactive fat mass typically seen – but also affects general activity levels (e.g. Kelley et al., 2025, and references therein). Because of either relative confinement or reduced foraging requirements, many species typically reduce activity levels in captivity (Mason, 2010; Glazier, 2020; Glazier and Gjoni, 2024). Although metabolically inactive, increased fat deposition can increase energy expenditure through a higher cost of transport (e.g. Ewart et al., 2022). However, the reduced activity levels mean that this increased cost of transport is unlikely to be a significant contributing factor to the daily energy expenditure in captive animals.

The assessment of metabolic rate, and particularly BMR, is conducted under specific restrictive conditions (i.e. of postabsorptive, adult, resting, non-breeding animals, assessed in their respective circadian rest phase and within their thermoneutral zone; McNab, 1988, 1997, 2012), which enables like-for-like comparisons to be made between individuals, populations and species. However, one aspect often overlooked is the possible effect of (a) bringing a species into captivity from the wild and (b) mixing and comparing the metabolic rates of recent wild-caught animals with those of longstanding captive animals. While it has been shown that metabolic rate is plastic and can change in response to an animal's internal state or environmental constraints (Norin and Metcalfe, 2019), acclimation to a captive environment – from a formerly wild environment – prior to metabolic assessment is a factor that often appears variable between studies. For example, there is considerable interstudy disparity in the lengths of time that African mole-rats were maintained in captivity before metabolic rates were assessed (Table 1 and references therein). In some studies, metabolic rate has been assessed in mole-rats kept in captive conditions for only a few weeks, whereas other studies assessed animals that had been maintained in captivity for months or years or used captive-born animals. Because of their subterranean lifestyle, mole-rats do not exhibit a strong circadian rhythm, with regards to either a strict nocturnal or a diurnal cycle (Oosthuizen and Bennett, 2022). As such, ensuring measurements of metabolic rate adhere strictly to BMR requirements is not possible. Therefore, the majority of studies that measure metabolic rate in mole-rats define these measurements as RMR, despite all other requirements for BMR being met (i.e. thermoneutrality, non-breeding, post-absorptive) (e.g. Genoud et al., 2018). A recent review intimated that such differences in acclimation to a captive environment influence the RMR, at least in some species, resulting in decreased RMR in individuals acclimated for a longer period (Šumbera, 2019). Studies often use captive animals, yet the effects of captivity and, moreover, the length of acclimation to captivity on metabolic rate have not been identified. This raises questions as to whether animals can metabolically acclimate over a period of time to an environmental stressor (e.g. captivity) and, if true, whether it is then appropriate to compare metabolic rates of populations that have acclimated to different conditions (such as comparing wild and captive populations).

**Table 1.** A breakdown of metabolic studies of African mole-rats, grouped by the respective length of acclimation to captivity prior to metabolic assessment

Length of acclimation (months)	African mole-rat species	Reference
<2	*Cryptomys hottentotus hottentotus*	Bennett et al., 1992
	*Cryptomys hottentotus mahali*	Broekman et al., 2006
	*Cryptomys hottentotus natalensis*	Bennett et al., 1993a
	*Cryptomys hottentotus nimrodi*	Bennett et al., 1996
	*Cryptomys hottentotus pretoriae*	Haim and Fairall, 1986
	*Fukomys damarensis*	Bennett et al., 1992
	*Heliophobius argenteocinereus*	McNab, 1966
	*Heterocephalus glaber*	McNab, 1966
2–3	*Bathyergus janetta*	Lovegrove, 1986a
	*Bathyergus suillus*	Lovegrove, 1986a
	*Cryptomys natalensis*	Bennett et al., 1993a
	*Fukomys anselli*	Bennett et al., 1994
	*Fukomys bocagei*	Bennett et al., 1994
	*Fukomys damarensis*	Bennett et al., 1992
	*Fukomys darlingi*	Bennett et al., 1993b
	*Fukomys mechowii*	Bennett et al., 1994
	*Georychus capensis*	Lovegrove, 1987
3–12	*Cryptomys hottentotus*	Bennett et al., 1992
>12	*Fukomys anselli*	Marhold and Nagel, 1995
	*Fukomys damarensis*	Lovegrove, 1986b
	*Fukomys darlingi*	Zemanová et al., 2012
	*Heliophobius argenteocinereus*	Zelová et al., 2007
	*Heterocephalus glaber*	Buffenstein and Yahav, 1991

Data are from Šumbera (2019).

With the exception of two published studies (Bennett et al., 1992, 1993a) that assessed the effect of metabolic acclimation to captivity in three African mole-rats (*Cryptomys hottentotus natalensis, Cryptomys hottentotus hottentotus and Fukomys damarensis*), the metabolic response to being brought into captivity remains largely unexplored. These studies demonstrated that both *C. h. natalensis* and *C. h. hottentotus* exhibited a considerably reduced mass-specific resting metabolic rate (msRMR); the msRMR of *C. h. natalensis* decreased by 22.3% after 2 months, while that of *C. h. hottentotus* decreased by 33.3% over a similar period. By contrast, however, *F. damarensis* exhibited a 4.6% increase in mean msRMR, following a 2 month acclimation to captivity. We note here that for a considerable amount of time, mass-correcting metabolic rate (i.e. dividing RMR by body mass) to account for differences between species was commonplace (Tschöp et al., 2011).

Three explanations were proposed to explain why the two *Cryptomys* species exhibited this reduction in their msRMR when acclimated to captive conditions, while also speculating why *F. damarensis* did not exhibit a similar reduction (Bennett et al., 1992). Firstly, the associated stress of capture and subsequent maintenance of the mole-rats under laboratory conditions may have increased their RMR. Despite it being suggested that all three species experienced comparable post-capture stress on account of an equivalent methodological approach, differences in the duration of transport and the colonies' sociodemographic may have induced different post-capture stress levels. Furthermore, there is likely to be interspecific variation in how stress is perceived. More specifically, the *Cryptomys* subspecies may have exhibited a heightened stress response immediately following capture (Hart et al., 2022), elevating their baseline metabolic rate (i.e. post-capture RMR), thus giving the impression of a greater reduction in their RMR when reassessed following a period of acclimation under captive conditions, compared with *F. damarensis* (Medger et al., 2018). Secondly, RMR may also have decreased in captivity because of reduced foraging. In the wild, animals are required to forage (i.e. move and tunnel) for irregularly distributed food resources, which maintains body musculature. It is possible that changes in body composition – a relative increase in body fat, which is metabolically inactive, coupled with reduced musculature – in captive animals that are fed *ad libitum* and have restricted movement may have reduced RMR. Thirdly, the depth of the nesting chamber in naturally occurring colonies was suggested as the most feasible explanation for the observed metabolic acclimation (Bennett et al., 1992). It was explained that wild *C. h. hottentotus* and *C. h. natalensis* have nests at considerably shallower depths (40 cm) than those of *F. damarensis* (1.6–2.3 m) (Bennett et al., 1992, 1993a), which despite being largely buffered against above-ground climatic conditions, still likely experience greater seasonal temperature fluctuations. Mole-rat species that nest at shallower depths experience greater selective bioclimatic variability, and thus in response, have a greater physiological plasticity (Bennett et al., 1992, 1993a). Furthermore, it is worth noting an additional explanation; the observed increase in msRMR in *F. damarensis* could potentially be attributed, at least in part, to an insufficient acclimation period. While these are convincing explanations, the body mass at capture and following the respective periods of acclimation to captivity were not detailed in the two previous studies (body mass was not reported independently from msRMR), and thus the potential effects of body mass changes cannot be determined.

This study set out to explore whether another *Cryptomys* subspecies, *Cryptomys hottentotus pretoriae*, similarly exhibits a reduction in their msRMR following a period of time maintained under captive conditions and, if so, whether this could, in part, be driven by suspected increases in body mass when brought into, and maintained within, a captive environment. Secondary aims of the study were to determine whether differences exist in the RMR, body mass, msRMR and respiratory quotient (RQ) of animals 7 days post-capture and animals maintained in captivity for 12 months. *Cryptomys h. pretoriae* do not exhibit a strict dominance hierarchy, nor can members of a colony be differentiated into distinct working groups based on body mass, thus inferring that all members work equally (Moolman et al., 1998). This equal contribution to colony maintenance is believed to have resulted in a reduction of body mass dimorphism (Süess et al., 2023). Despite this, determining to what extent sex explains variation in RMR, body mass, msRMR and RQ may distinguish physiological differences that may not otherwise be identified.

## MATERIALS AND METHODS

### Study animals

Fifteen *Cryptomys hottentotus pretoriae* Roberts 1913 from three colonies were trapped under license using live Hickman traps (Hickman, 1979), in the suburb of Tygerpoort (25°47′S, 28°21′E), Pretoria, South Africa. Animals were caught over a period of approximately 3 days, in which the traps were checked and maintained regularly. Mole-rats were transferred to the Department of Zoology and Entomology at the University of Pretoria (UoP), and housed in large plastic containers. Throughout this study, animals were provisioned with appropriate nesting material and were fed *ad libitum* on sweet potatoes, which were replaced daily. The animals were maintained in large polyurethane containers, housed in a climate-controlled laboratory that maintained an ambient temperature (*T*_a_) of 23–5°C, a relative humidity of 40–60% and a light cycle set to 12 h light:12 h dark (Ivy et al., 2020). Experimental procedures involving live animals and data collection described herein were approved by Royal Holloway University of London and the UoP Animal Ethics Committee (Ref. EC004-19). The study was conducted in accordance with appropriate institutional and national guidelines. From here on in, ‘wild’ refers to 7 day-acclimated mole-rats and ‘captive’ means 12 month-acclimated mole-rats.

### Experimental procedure

RMR was determined through the measurement of the rate of oxygen consumption (*V̇*_O_2__) and carbon dioxide production (*V̇*_CO_2__), using an open-flow respirometer (Sable Systems International, Las Vegas, NV, USA). Despite an apparent absence of circadian metabolic rhythms among these species (Bennett and Faulkes, 2000), for continuity with other metabolic studies on African mole-rats and to follow established protocols, we conducted all assessments between 08:00 h and 18:00 h, to mitigate against the potential effects of endogenous metabolic rhythms. An absence of circadian metabolic rhythms, along with the unknown extent of stress and variation in the time that animals were fasted, meant that RMR, rather than BMR, was a more applicable measure of energy expenditure in this study; the strict criteria for BMR could not be guaranteed (Šumbera, 2019).

Each respirometry assessment lasted approximately 65 min and consisted of a 10 min baseline to assess ambient O_2_ level, a 45 min metabolic assessment, followed by a further 10 min baseline to reassess ambient O_2_. The respirometer consisted of an airtight 2.5 l (19.2 cm long×15.2 cm wide×8.8 cm high) acrylic container, fitted with 4 mm inlet and outlet ports. Outside air was pulled through the respirometer at a flow rate of 600 ml min^−1^, resulting in a flush-out rate of approximately 4 min 10 s. The analogue outputs of O_2_ (%), CO_2_ (%), flow rate (ml min^−1^), relative humidity (%), barometric pressure (kPa) and temperature (°C) were recorded concurrently using a universal interface (UI2, Sable Systems International). These measurements were sampled (1 Hz) and monitored in real-time using ExpeData software (Sable Systems International), which enabled the progress and stability of each animal's respirometry trace to be visually assessed. Additionally, this enabled the manual addition of markers on the trace to note times of aberrant behavioural observations or external confounding factors. This real-time monitoring also safeguarded against potentially dangerous spikes in CO_2_ or drops in O_2_, at which point the assessment would have been terminated. Body mass (g) was measured immediately preceding each assessment using Oertling electronic weigh scales.

Incurrent airflow was controlled using a flow regulating pump (SS-4, Sable Systems International), calibrated against a certified mass flow meter (FoxBox, Sable Systems International), placed downstream of the respirometry chamber. Fractional concentration of O_2_ was measured using an oxygen analyser (FC-10a, Sable Systems International), which was calibrated to ambient air O_2_ concentration (20.95%) before each trial. Fractional concentration of CO_2_ was measured using a CO_2_ analyser (CA-10a, Sable Systems International), and relative humidity was measured using a water vapour analyser (RH-300, Sable Systems International). Barometric pressure and temperature were measured from inbuilt sensors in the FC-10a oxygen analyser. Anhydrous Indicating Drierite™ was used to scrub atmospheric water from the excurrent air between the water vapour and CO_2_ analysers, and again between the CO_2_ scrubber and the oxygen analyser (W. A. Hammond Drierite Company Ltd). CO_2_ was scrubbed from the excurrent air between the CO_2_ and O_2_ analysers (Soda Lime, Sigma-Aldrich, Merck KGaA, Darmstadt, Germany).

Data, once exported from ExpeData, were processed in Matlab (v9.6, The MathWorks Inc. 2019, Natick, MA, USA). O_2_ and CO_2_ were corrected for baseline drift and any time lag between these two variables (due to the delay in airflow between analysers) was corrected using cross-correlation. The fractional O_2_ signal was corrected for the removal of CO_2_ (O_2,corrected_), the fractional CO_2_ signal was corrected for the removal of water vapour (CO_2,corrected_) and the flow rate was corrected to standard temperature and pressure (STP) conditions. A 5 min minimum analysis region was selected for RMR, corresponding to the lowest stable O_2_ consumption and CO_2_ production, during which the animal was considered to be most restful (see also Merchant et al., 2024b; Thirkell et al., 2025). The average over this period was used to obtain RMR estimates (*V̇*_O_2__ and *V̇*_CO_2__), calculated using the formulae:
(1)

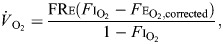
and
(2)

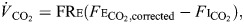
where *F*i and *F*e are incurrent and excurrent fractional concentrations (%) of O_2_ and CO_2_ (Lighton, 2008). The ratio of *V̇*_CO_2__ to *V̇*_O_2__ determined the RQ (Lighton, 2008). *V̇*_O_2__ is presented as the mean±s.d., corrected to STP conditions.

Historically, the approach taken in many studies has been to report msRMR. We recognise that mass-correcting metabolic rates may now be considered too simplistic an approach, as species with large body masses tend to exhibit reduced msRMR. However, the results of this study were being compared with two similar historic studies that reported msRMR, where neither whole-animal RMR nor body mass was reported. It was for these reasons that we present and discuss msRMR values.

Metabolic assessments were undertaken 7 days post-capture (‘wild’) and repeated on the same animals approximately 12 months later (‘captive’). Animals were fasted for >12 h prior to RMR assessments, to ensure a post-absorptive state and exclude the potential influence of digestion on metabolic activity (Šumbera, 2019; Wallace et al., 2021). Only adult animals (>50 g) that were considered to be neither pregnant nor lactating were assessed (N.C.B., personal observation). Three animals on capture were considered juveniles and were omitted from the unacclimated assessment; however, they were later included in the acclimated assessment, having all exceeded 50 g.

The RMR (ml O_2_ h^−1^) of 11 wild-caught and 15 captive *C. h. pretoriae* was measured (Table 2). One further wild individual was assessed, but was identified to be highly influential and a statistical outlier (*D*≥0.5) and was subsequently omitted from statistical analyses. Individuals could not be distinguished following acclimation to captivity, which precluded repeated measures analysis. As individuals were indistinguishable, this violates the non-independence assumption of both GLM and ANOVA analyses. As such, statistical analyses should be interpreted with caution, as it is not possible to determine the effect of repeatability.

**Table 2.** The mean resting metabolic rate (RMR), body mass, mass-specific (ms)RMR and respiratory quotient (RQ) of a population of wild and captive *Cryptomys hottentotus pretoriae*

	Wild (*N*=11)		Captive (*N*=15)	
	Male (*N*=6)	Female (*N*=5)	Male (*N*=7)	Female (*N*=8)
RMR (ml O_2_ h^−1^)	107.23±16.22		100.88±17.73	
	106.08±17.53	112.40±10.36	103.73±22.07	98.37±14.00
Body mass (g)	79.64±22.19		102.00±22.20	
	77.44±20.88	77.80±22.95	118.00±15.25	88.00±17.50
msRMR (ml O_2_ g^−1^ h^−1^)	1.41±0.33		1.04±0.32	
	1.43±0.33	1.33±0.40	0.88±0.11	1.18±0.37
RQ	0.74±0.12		0.66±0.05	
	0.74±0.10	0.76±0.26	0.64±0.03	0.67±0.06

The data have been further stratified by sex. Individuals were indistinguishable, thus violating the non-independence assumption of both GLM and ANOVA analyses.

### Statistical analyses

Data (see Tables S1 and S2) were analysed in R v4.4.1 using linear models, to determine whether acclimation to captivity explained variation in the assessed RMR, body mass, msRMR and RQ of *C. h. pretoriae*. The model for RMR included body mass as a continuous predictor, with captivity (captive or wild) and sex (male or female) as categorical fixed effects, as well two- and three-way interactions between body mass, captivity and sex. The models for body mass and msRMR included captivity and sex, as well as their interaction. Interaction terms were sequentially removed when non-significant (*P*>0.05). Where interactions were significant, pairwise *post hoc* comparisons were undertaken using the Tukey method implemented in the R package ‘emmeans’ v1.10.6 (https://CRAN.R-project.org/package=emmeans).

## RESULTS

### RMR

**Fig. 1.**
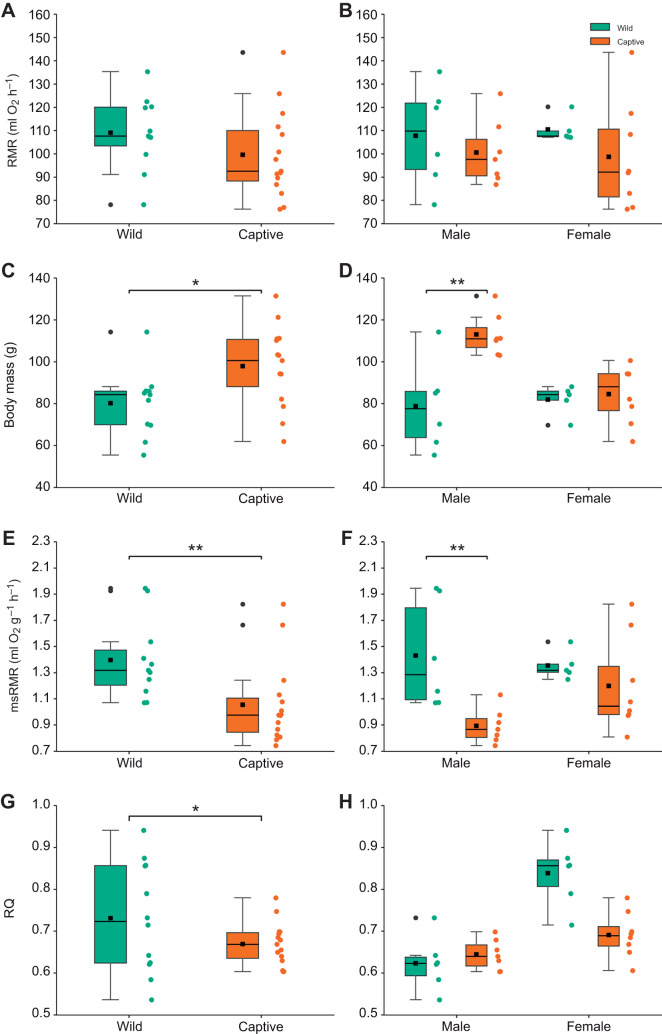
**Metabolic rates of captive and wild *Cryptomys hottentotus pretoriae*.** The difference in (A) resting metabolic rate (RMR), (C) body mass, (E) mass-specific resting metabolic rate (msRMR) and (G) respiratory quotient (RQ) between a wild (*N*=11) and captive (*N*=15) population of *C. h. pretoriae*. (B,D,F,H) Differences in RMR, body mass, msRMR and RQ, respectively, stratified by sex. Metabolic rate was assessed within the known thermoneutral zone (30–36°C) of their closest relative, *Cryptomys hottentotus mahali*. Box plots show medians, upper and lower quartiles and 1.5× the interquartile range. Mean values are represented by a black solid square, while non-significant outliers are represented by a black solid circle. Asterisks indicate statistical significance (*P<0.05, **P<0.01). Individuals were indistinguishable, thus violating the non-independence assumption of both GLM and ANOVA analyses.

There were no significant two- or three-way interactions between body mass, captivity and sex in the model for RMR (*P*>0.69 in all cases). There was no significant difference (*t*_22_=1.44, *P*=0.16; Fig. 1A) in the RMR of wild (107.23±16.22 ml O_2_ h^−1^) and captive animals (100.88±17.73 ml O_2_ h^−1^). The effect of sex was also not significant (*t*_22_=−0.29, *P*=0.77; Fig. 1B).

### Body mass

There was a significant sex by captivity interaction in the model for body mass (*t*_22_=−2.82, *P*=0.01; Fig. 1C). *Post hoc* tests revealed that there was no significant difference in mean body mass between wild and captive females (*t*_22_=0.33, *P*=0.99; Fig. 1D), but males had a significantly higher mean body mass (*t*_22_=4.37, *P*=0.001; Fig. 1D) after 12 months in captivity.

## DISCUSSION

Our results show there was no significant difference in the RMR of *C. h. pretoriae* assessed 7 days post-capture (wild) and again after being maintained in captivity for 12 months (captive), nor was sex a significant determinant of RMR (Fig. 1A,B). Despite this, and similar to conclusions for *C. h. natalensis* and *C. h. hottentotus*, *C. h. pretoriae* exhibited a significant reduction in msRMR of 26.1% over a 12 month period of captivity (Fig. 1E). Interestingly, the RMR of captive and wild animals was not significantly different, despite the captive animals weighing significantly more (Fig. 1A,C). Thus, it is likely that the animals maintained in captivity for 12 months gained body fat. While an increase in body fat increases an animal's cost of transport (Ewart et al., 2022; Halsey and White, 2019), at rest, which the animals were during the metabolic assessment, fat is relatively metabolically inactive (Aarseth et al., 1999; Goyal et al., 1981; Rea and Costa, 1992).

It was previously suggested that mole-rat species that nest at shallower depths in the wild exhibit greater physiological plasticity as a result of experiencing greater bioclimatic variation, which is the determining driver of reduced msRMR (Bennett et al., 1992, 1993a). However, the current study suggests that the observed reduction in msRMR is instead principally driven by a significant increase in mean body mass; captive animals were 28.1% heavier than wild animals. Another interesting facet is that when sex is accounted for, *C. h. pretoriae* are considered to be one of the least sexually dimorphic *Cryptomys* subspecies (Süess et al., 2023); indeed, the mean body mass of wild male (77.44±20.88 g; *N*=6) and female (77.80±22.95 g, *N*=5) animals is comparable. However, the effect of captivity on body mass differed between sexes; males increased their body mass by 52.4%, compared with females, which increased their body mass by 13.1% (Fig. 1D,F). Haupt et al. (2017) found that there was no significant difference in activity levels between males and females at a variety of ambient temperatures, measured during the dark period of their daily cycle. During the light period, males were more active at 30°C and 25°C while females were significantly more active at 20°C. Given the mole-rats in our study were kept at 23–25°C, it is possible that females were slightly more active at the lower end of the ambient temperature range, resulting in males gaining body mass in captivity, while the females did not. Understanding the mechanisms that underpin the observed differences in response to captivity between the different mole-rat species would be fruitful. McKechnie et al. (2006) demonstrated that the effect of captivity on BMR in birds was body-mass dependent, with lighter species exhibiting a dramatic increase in BMR in response to captivity, while heavier species demonstrated a significant decrease (see also Mansour, 2005; Thompson et al., 2015; Minnaar et al., 2014). The body mass range in the McKechnie et al. (2006) study was 5–4000 g, which is substantially greater than the 35–550 g mass range for the species of mole-rats in the captive comparison studies. Nevertheless, determining how elements of species-specific physiology and behaviour interact with metabolic rate would be worthwhile.

Under the relatively stable conditions of captivity, it is indicative that males can reach considerably greater body mass than females, to the point where this species does exhibit sexual size dimorphism. Furthermore, this infers that there are selective pressures acting on wild males that prevent them attaining a greater body mass than wild females. We posit that in the wild there is a trade-off between burrow size and predator intrusion, which may limit the diameter of burrows for wild colonies and thus limits the body mass that individuals can reach. The cross-sectional area of a burrow scales in proportion to the size of the animal that constructed it as mass^∼2/3^ (White, 2005), so the energetic cost of making and maintaining a larger burrow is greater for larger animals (Vleck, 1979; White, 2001; White et al., 2006). In addition, larger *C. h. natalensis* males are more at risk of exercise-induced hyperthermia (Jacobs et al., 2022; Finn et al., 2022) even though they dwell in a relatively cool environment in comparison to *C*. *h. pretoriae* (Süess et al., 2023). As such, the body mass of male C. *h. pretoriae* may be limited by exercise-induced hyperthermia in their warm semi-arid environment. Despite *C. h. pretoriae* being considered to exhibit an equal division of labour, which might imply no distinct hierarchical dominance differences between the sexes, males tend to be more dominant in captivity (Moolman et al., 1998), and thus may consume more purely through preferential access to food.

Captivity can cause body mass to increase in animals, as a result of the relative reduction in activity levels and constant regular supply of food (Fischer and Romero, 2019; Speakman and Hall, 2023). In 17% of studies (6 of 36), wild animals gained mass above their starting condition on initial introduction into captivity. For instance, North Island saddlebacks (*Philesturnus rufusater*) experienced mass loss on their first day in captivity; however, by the third day, they had not only recovered the lost mass but also surpassed their original capture mass (Adams et al., 2011). Sex-specific body mass changes in response to captivity have not been detailed in many studies. In captive golden-mantled ground squirrels (*Spermophilus lateralis*), males stored more fat than females (Blake, 1972), regardless of the origin of the populations (e.g. high or low altitude), akin to the males in the current study. In a study looking at captive lemurs from nine different species, there was only one example in which males were heavier than females after a period in captivity (Terranova and Coffman, 1997). A long-term study which tracked physiological and behavioural changes in cavies (*Cavia tschudii*) from capture in the wild to 30 generations in captivity found no significant changes in most key behavioural or hormonal stress responses (Künzl et al., 2003). The only behavioural difference was a reduction in explorative behaviour in the captive animals, but this was not sex specific, and thus is unlikely to explain the differences between the sexes we observed. That male mole-rats would show such an increase in body mass in response to captivity contrasts with prior studies on rodents. Speakman and Hall (2023) found no evidence that wild small rodents brought into captivity developed obesity, even when exposed to high-fat diets. The authors suggested that this was due to rodents typically being a prey species, and the selection pressures to maintain manoeuvrability and escape speed. It is feasible that the largely fossorial lifestyle of Bathyergidae reduces potential predation pressure, which in turn means they have an atypical body mass response, in males at least, to captivity.

**Fig. 2.**
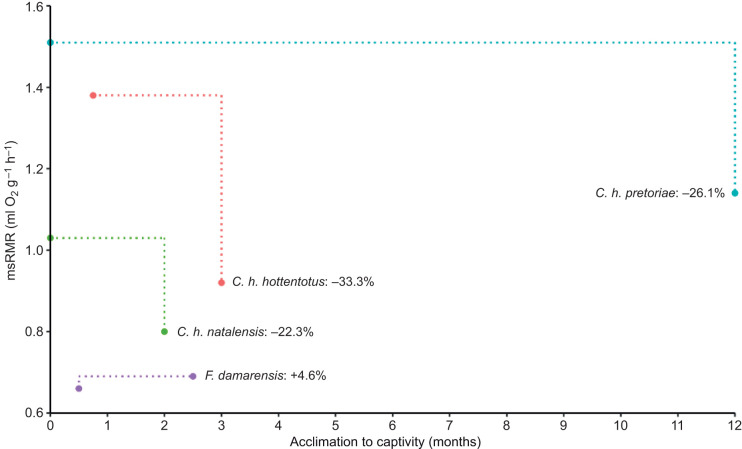
**Comparison of msRMR of four wild-caught populations of African mole-rat species post-capture and again following acclimation to captivity.** Data are shown for *C. h. pretoriae* (this study), *Cryptomys hottentotus hottentotus* (Bennett et al., 1992), *Cryptomys hottentotus natalensis* (Bennett et al., 1993a) and *Fukomys damarensis* (Bennett et al., 1992).

It is this significant increase in male body mass that is likely to be driving the significant difference in msRMR identified between wild and captive animals; animals 7 days post-capture had a significantly greater msRMR than animals maintained in captivity for 12 months. While sex had no effect on msRMR, there was a decrease in the mean msRMR of both captive males and females. *Cryptomys* subspecies (*C. h. hottentotus*, *C. h. natalensis* and *C. h. pretoriae*) all exhibit a marked reduction in their msRMR with respect to time in captivity (Fig. 2). What is unknown is whether this reduction in msRMR of acclimated individuals is a genus-specific response to captivity, or whether this physiological response characterises all African mole-rat species (Bathyergidae), or indeed all mammal species, following a period of acclimation. Whilst this study supports previous findings that African mole-rats require a period of time to metabolically acclimate to captive conditions, neither the rate of metabolic acclimation to captivity (i.e. exponential decay or sigmoid decay) nor the time needed for this to occur (i.e. 1 month, 3 months, 5 months, etc.) has been specifically evaluated. This, along with temporal changes in body composition should be the focus of future research.

Furthermore, there were marked differences in the RQ of wild and captive animals, despite the fact that they were food deprived for the same period prior to experiments; wild animals had a significantly greater mean RQ than captive animals (Fig. 1G). Despite a reduction in the mean RQ of both males and females, no significant effect of sex was identified (Fig. 1H). Four wild animals were identified to have an RQ greater than 0.8, which might imply that these individuals were not post-absorptive, despite all animals being fasted for at least 12 h. Mole-rats are typically considered to be post-absorptive after 3 h (Šumbera, 2019), and brown rats (*Rattus norvegicus*) fed formulated diets are post-absorptive within 7.5 h (Secor, 2009). But the specific time required to achieve a post-absorptive state has not been experimentally determined for most species (Genoud et al., 2018), including mole-rats. If the wild animals were post-absorptive, then the elevated RQ might arise if they were voluntarily underfeeding during the initial 7 days in captivity, resulting in an increase in the preponderance of protein-over-fat breakdown as their fat stores were expended during the initial days in captivity. Such a pattern of rising RQ from ∼0.72 to ∼0.8 has been observed over a similar time scale in rats during starvation (Kleiber, 1961). An alternative explanation is that the mole-rats were catabolising protein, particularly if they were lean upon capture, and then did not eat for the first day or two in captivity.

As previously suggested, a period of acclimation to captivity is necessary to facilitate comparable interspecies assessments in African mole-rats (Bennett et al., 1992, 1993a). Indeed, great care should be taken when comparing and interpreting metabolic studies of acclimated and unacclimated animals. It should be noted that it is likely that there is high interspecies variation in the rate of acclimation and, therefore, the rate of acclimation should ideally be assessed on a species-by-species basis. As important as it is not to compare BMR with RMR, it is similarly important not to compare unacclimated and acclimated metabolic rates. We suggest that analogous to assessing thermoneutral zones through assessments at incrementally increasing ambient temperatures, metabolic rate should be assessed at multiple time points post-capture. This would determine both the rate of metabolic acclimation and the acclimated metabolic rate. Moreover, such close monitoring of metabolic rate would also mitigate for the potential effects of high levels of individual variation in metabolic rate. Studies to date may have routinely underestimated many species' true metabolic rate by failing to account for physiological maladaptation to captivity. It would be considered best practice for all future studies to explicitly state whether their assessed metabolic values are from acclimated or unacclimated animals, as well being transparent as to the length of acclimation.

Metabolic rate, particularly RMR, is a widely used physiological marker of baseline energetic expenditure. The benefit of assessing RMR of any vertebrate species is that it is assessed under standard conditions (i.e. post-absorptive, adult, non-reproductive, non-active and during the species' natural rest phase), with BMR assessed under even stricter conditions. What we have shown is that the msRMR of acclimated animals can be in excess of 25% lower than that of unacclimated animals and, therefore, great care needs to be taken to ensure that conclusions appropriately account for studies that have used a mix of acclimated and unacclimated animals.

Whereas wild animals likely contend with variable food availability, predation pressures, burrow climate (including ambient temperature, oxygen and carbon dioxide concentrations, soil hardness, etc.), which can vary daily and seasonally, these parameters are maintained relatively stable in captivity and animals are therefore not metabolically challenged to the same extent. Mole-rats also tend not to actively burrow in captivity; therefore, there is a further energetic saving. Energy can be redirected from maintaining body temperature, burrow maintenance and food acquisition to growth and reproductive fitness. Furthermore, the maintenance of captive animals with *ad libitum* access to food at a relatively constant ambient temperature coupled with their reduced foraging and activity may collectively explain the suspected increase in relative body fat. Fat is metabolically inactive and while there is a metabolic cost of moving a larger body mass (i.e. net cost of transport), this perhaps does not play such an integral part within a captive environment where movement is constrained, and food is typically provisioned continually.

### Conclusion

At the population level, we demonstrate that *C. h. pretoriae* exhibits a 26.1% reduction in msRMR after 12 months acclimation to a captive environment. Whereas it was previously suggested that nest depth is the most likely driver of reduced msRMR, the interpretation of this study is that this is principally driven by a significant increase in mean body mass; captive animals were 28.1% heavier than wild animals. We posit that this may be a typical response of formerly wild animals that have no predation pressure, and are maintained at a near-constant temperature, provisioned with food *ad libitum* – reducing the requirement to forage – and held under relatively constrained conditions that likely reduce physical activity and energy expenditure. Indeed, this may be characteristic of many mammalian species and has wider connotations for studies that have included interspecies comparisons. To this end, studies should explore body compositional changes in response to captivity.

The benefit of assessing RMR of any vertebrate species is that it is, in theory, assessed under like for like conditions. What we have shown is that the msRMR of captive animals can be in excess of 25% lower than that of recently wild-caught animals and therefore this raises questions as to whether it is appropriate to make comparisons without first accounting for metabolic acclimation. Studies to date may have routinely overestimated or underestimated many species' true metabolic rate by failing to account for acclimation to captivity (e.g. Fischer and Romero, 2019).

## Supplementary Material

10.1242/jexbio.252449_sup1 Supplementary information
