# Supplementary material for: Metabolic acclimation to captivity in highveld mole-rats (Cryptomys hottentotus pretoriae) is driven by sex-specific body mass increases
Source: J Exp Biol. 2026 May 19;229(10):jeb252449. doi: 10.1242/jeb.252449 (PMC13245895; doi:10.1242/jeb.252449)
Supplement: Supplementary information [file jexbio-229-252449-s1.pdf]

Table S1. Full data set from the captive (*N*=15) highveld mole-rats (*Cryptomys hottentotus pretoriae*). ID refers to each individual. Ms/MRMR is mass corrected resting metabolic rate (ml O_2 $g^{-1}.hr^{-1}$). RMR is resting metabolic rate (ml O_2 hr^{-1}). BM is body mass (g), and sex is either male (M) or female (F).

ID	ms/RMR	RMR	BM	SEX
1	0.7433	97.69	131.42	M
2	0.7875	86.85	110.28	M
4	0.8238	91.42	110.97	M
5	0.8679	89.71	103.37	M
6	0.9204	111.64	121.3	M
8	0.9778	100.82	103.11	M
12	1.1315	125.89	111.26	M
3	0.8086	76.24	94.29	F
7	0.9737	91.9	94.38	F
9	0.9827	92.57	94.2	F
10	1.0103	83.01	82.16	F
11	1.0773	108.37	100.59	F
13	1.6644	117.41	70.54	F
14	1.8242	143.6	78.72	F
15	1.2419	77	62	F

Journal of Experimental Biology • Supplementary information

Table S2. Full data set from the wild (*N*=11) highveld mole-rats (*Cryptomys hottentotus pretoriae*). ID refers to each individual. Ms/MRMR is mass corrected resting metabolic rate (ml O_2 $g^{-1}.hr^{-1}$). RMR is resting metabolic rate (ml O_2 hr^{-1}). BM is body mass (g), and sex is either male (M) or female (F).

ID	ms/RMR	RMR	BM	SEX
7	1.40988634	78.15	55.43	M
2	1.07037124	91.11	85.12	M
8	1.94498539	119.85	61.62	M
3	1.15901963	99.78	86.09	M
6	1.07185996	122.46	114.25	M
10	1.9254517	135.34	70.29	M
9	1.31888317	107.7	81.66	F
4	1.24892404	107.37	85.97	F
5	1.30277514	109.85	84.32	F
11	1.53562724	107.11	69.75	F
1	1.36533878	120.3	88.11	F

Journal of Experimental Biology • Supplementary information
